# Isolation, Physicochemical Properties, and Structural Characteristics of Arabinoxylan from Hull-Less Barley

**DOI:** 10.3390/molecules26103026

**Published:** 2021-05-19

**Authors:** Haoyingye Yao, Yuxiao Wang, Junyi Yin, Shaoping Nie, Mingyong Xie

**Affiliations:** 1 State Key Laboratory of Food Science and Technology, China-Canada Joint Lab of Food Science and Technology (Nanchang), Nanchang University, 235 Nanjing East Road, Nanchang 330047, China; ncuyaohaoyingye@email.ncu.edu.cn (H.Y.); yuxiaowang@email.ncu.edu.cn (Y.W.); yinjy@ncu.edu.cn (J.Y.); spnie@ncu.edu.cn (S.N.); 2 National R&D Center for Freshwater Fish Processing, Jiangxi Normal University, Nanchang 330022, China

**Keywords:** hull-less barley, arabinoxylan, structural characteristics

## Abstract

Arabinoxylan (HBAX-60) was fractioned from alkaline-extracted arabinoxylan (HBAX) in the whole grain of hull-less barley (*Hordeum vulgare* L. *var. nudum* Hook. f. *Poaceae*) by 60% ethanol precipitation, which was studied for physicochemical properties and structure elucidation. Highly purified HBAX-60 mainly composed of arabinose (40.7%) and xylose (59.3%) was created. The methylation and NMR analysis of HBAX-60 indicated that a low-branched β-(1→4)-linked xylan backbone possessed un-substituted (1,4-linked β-Xyl*p*, 36.2%), mono-substituted (β-1,3,4-linked Xyl*p*, 5.9%), and di-substituted (1,2,3,4-linked β-Xyl*p*, 12.1%) xylose units as the main chains, though other residues (α-Ara*f*-(1→, β-Xyl*p*-(1→, α-Ara*f*-(1→3)-α-Ara*f*-(1→ or β-Xyl*p*-(1→3)-α-Ara*f*-(1→) were also determined. Additionally, HBAX-60 exhibited random coil conformation in a 0.1 M NaNO_3_ solution. This work provides the properties and structural basis of the hull-less barley-derived arabinoxylan, which facilitates further research for exploring the structure–function relationship and application of arabinoxylan from hull-less barley.

## 1. Introduction

Hull-less barley (*Hordeum vulgare* L. *var. nudum* Hook. f. *Poaceae*; “Qingke” in Chinese), also known as highland barley, is mainly grown in the Qinghai–Tibet Plateau. Since hull-less barley is rich in nutritious ingredients and offers some advantages for food uses, it has gradually been noticed over the past decade that hull-less barley is healthy and can be used as a functional food [1]. The limited value of nutrient compositions in traditionally processed barley products is underestimated and able to satisfy demand from people [2]. Arabinoxylan (AX) is one of the most special and valuable compounds in hull-less barley. It usually originates from the starchy endosperm and bran, and it has been studied as a natural interfacial antioxidant in emulsion [3], as well as for the interaction with gluten [4]. However, AX has not received much attention due to its isolation method and complex structure.

The substitution of cereal AX is α-L-arabinofuranosyl (Ara*f*) units occurred at the *O*-2 and/or *O*-3 sites of the β-(1→4)-D-xylopyranosyl (Xyl*p*) backbone [5]. Other substituents, such as glucuronosyl residues and short oligosaccharide side chains, are also present [6,7]. The structural characteristics of AXs are related to the used raw material and various extraction methods, such as hot water, alkaline solutions, diluted acids, and various combinations of the techniques. For example, corn hull-derived AX contains a higher content of galactose or glucuronic acid than barley AX, while AX extracted by enzyme has a lower molecular mass (*M*_w_) value than an alkali-extracted fraction [8,9]. Physical treatments, such as extrusion cooking, would break glycosidic bonds and reduce the *M*_w_ of AX while lowering the degree of polymerization [10]. The *M*_w_ of AX was found to be positively correlated with the inhibition of starch digestibility [11], as well as viscosity, in vitro. The low ratio of arabinose to xylose (A/X) and unsubstituted parts of AXs, along with the cross-linking effect of the bound ferulic acid, were found to exert a negative effect on solubility [12]. Moreover, barley-derived AX has gained much more popularity regarding its biological activities, such as immunomodulation [13], glucose metabolism improvements [14], and the regulation of lipid metabolism [15]. In summary, structural characteristics (such as molecular masses and sugar repeating units) might result in different physicochemical properties, surface morphologies, rheological properties, and biological activities.

Due to its special geographical position and growing environment, the structural characterization, physicochemical properties, and solution properties of AX from whole hull-less barley grain have yet to be elucidated in depth. In this study, arabinoxylan (HBAX) was prepared from the whole grain of hull-less barley. Then, HBAX-60 was prepared from HBAX by 60% ethanol precipitation. Combined with the yield, monosaccharide composition, and molecular mass distribution, HBAX-60 was subjected to physicochemical analysis and structural elucidation in this study.

## 2. Results and Discussion

### 2.1. Physicochemical Properties

Initially, the enzymatic hydrolysis of lichenase was applied for HBAX purification. Due to the heterogeneous average molecular mass distribution detected by high performance gel permeation chromatography (HPGPC), it was not suitable for structural identification. Thus, 60% ethanol precipitation was used to purify HBAX to give a satisfying result in molecular mass profile, illustrating the high homogeneity of the purified fraction, which was named HBAX-60.

Table 1 lists chemical compositions of the hull-less barley AXs before and after purification. The main component of HBAX-60 was neutral sugar, the content of which was up to 98.3% after purification. Compared with the raw material, HBAX-60 had lower contents of starch and protein, which indicated that enzymatic hydrolysis and ethanol precipitation were effective in the extraction process for removing the starch and protein. Moreover, the content of total AX detected by the orcinol–hydrochloric acid method was increased after purification, while the β-glucan content analyzed by Megazyme β-glucan assay kit was decreased. HBAX-60 was composed of arabinose (40.7%) and xylose (59.3%), accompanied by a trace of glucose. To provide more information on the exact branching types and degrees, A/X was used as an indicator of a fine molecular structure [16]. The value of A/X in HBAX-60 was 0.7, representative of low-branched HBAX-60.

### 2.2. SEM Images and FT-IR Spectroscopy Analysis

**Figure 1:**
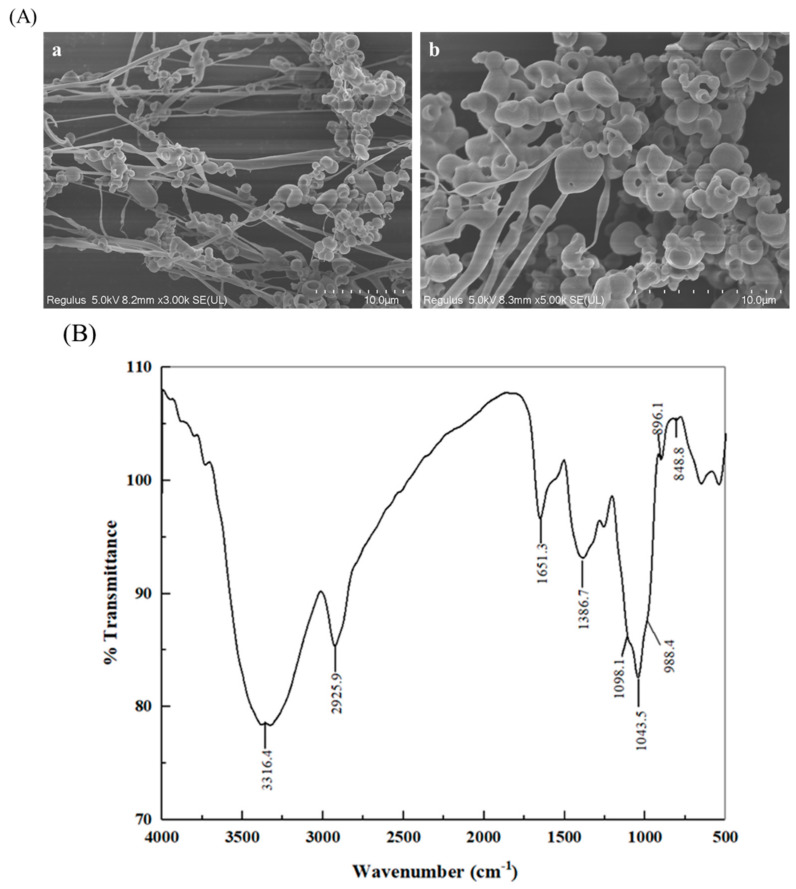
The results of SEM imaging and FT-IR spectroscopy: (**A**) SEM images at magnifications of (**a**) 3000× and (**b**) 5000×; (**B**) FT-IR spectrum of HBAX-60.

SEM images of HBAX-60 with different magnifications (3000 and 5000 fold) are shown in Figure 1A. Obviously, the microstructure of HBAX-60 was a little different from that of HBAX (Figure S1), the surface morphology of which comprised lamellar structures intertwined with linear and spherical shapes. After purification, HBAX-60 presented a spherical-like structure with some filamentary or linear structures. 

As shown in Figure 1B, an obvious shoulder peak (988.4 cm^−1^) and a comparatively intense peak (1043.5 cm^−1^) were typical for the FT-IR spectrum of HBAX-60 [17], which was similar with that of HBAX (Figure S2). Moreover, the characteristic peaks in the “fingerprint region” at 896.1 and 848.8 cm^−1^ were recognized as the β-pyranose and α-furanose of HBAX-60, respectively [18]. Additionally, two relatively extensive absorptions at 3316.4 and 2925.9 cm^−1^ were indicated as the vibration of O-H stretching group and the bending vibration of methylene groups, respectively. The peak at 1386.7 cm^−1^ was related to a deviational vibration of the C-H moiety. The absorption band centered at approximately 1650 cm^−1^ was attributed to the bound water.

### 2.3. Methylation Analysis

To identify linkage patterns, HBAX-60 was reduced to get its corresponding partially methylated alditol acetates (PMAAs) after methylation and hydrolyzation, followed by analysis by GC–MS [19]. Along with the monosaccharide composition, the results are shown in Table 2. Glycosyl linkages were characterized based on retention time and compared with the mass spectra of PMAAs (Figure S3). HBAX-60 mainly consisted of Ara*f*-(1→, →4)-Xyl*p*-(1→, →3,4)-Xyl*p*-(1→ and →2,3,4)-Xyl*p*-(1→ residues, accounting for 88.6% of the total proportion of sugar residues. Previous reports [6,20] has proposed the backbone of HBAX-60 as (1→4)-linked xylan consisting of un-substituted (36.2%), mono-substituted (*O*-2 or *O*-3, 7.1%), and di-substituted (*O*-2,3, 12.1%) Xyl*p* residues. The high proportion of Ara*f*-(1→ (34.4%) suggested that the terminal Ara*f* residues possibly appended to the backbone, mostly and directly as single chains. This AX derived from the proportion of ramified positions in the backbone had an accurate degree of substitution of 0.7 (Table 2), further confirming the results of monosaccharide compositions in Table 1. In general, HBAX-60 was considered to be a low branched arabinoxylan. The low branch of AX may be relevant to its low water-holding capacity and intrinsic viscosity [21], which could be proved and improved upon in further research.

### 2.4. NMR Analysis

**Figure 2:**
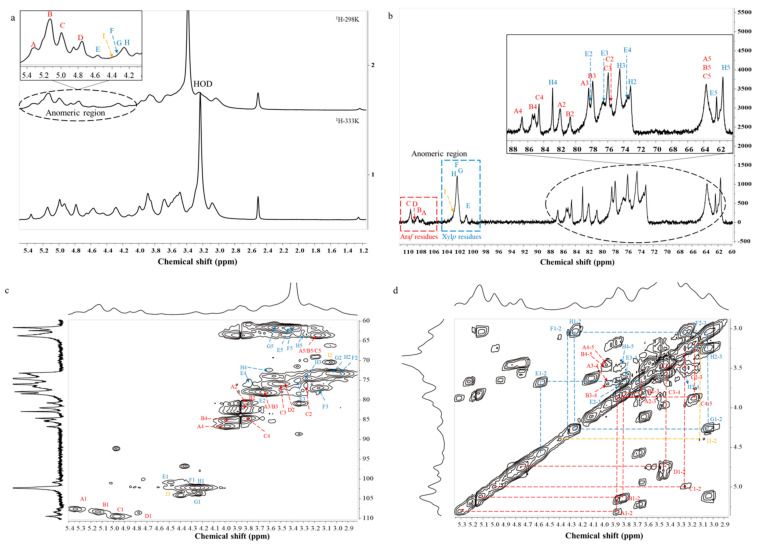
NMR spectra of HBAX-60: (**a**) ^1^H NMR spectrum, (**b**) ^13^C NMR spectrum, (**c**) HSQC NMR spectrum, and (**d**) COSY NMR spectrum.

NMR spectroscopy is one of the most essential and useful techniques for the structural identification of polysaccharides, including 1D NMR (^1^H and ^13^C NMR) and 2D NMR (COSY, TOCSY, NOESY, HSQC, and HMBC NMR). The ^1^H NMR spectrum (Figure 2a) of HBAX-60 was collected at 303 K, and signals were assigned by referring to previous data and methylation results. Nine explicit sugar residues are labelled in Figure 2a. The chemical shifts of anomeric protons in α-configuration ranged from 4.7 to 5.5 ppm, while the resonances observed at 4.2–4.7 ppm were derived from β-anomeric protons [20]. As the negative peaks were overlapped by an HOD peak at 3.39–3.59 ppm, another ^1^H NMR test (Figure 2a) was carried out at 333 K. 

Specifically, the H/C signals of α-Ara*f*-(1→ could be classified as three kinds of patterns, since α-Ara*f*-(1→ was attached to mono-substituted β-Xyl*p* residues and di-substituted β-Xyl*p* residues at the *O*-2 and/or *O*-3 sites [6,22]. Their anomeric proton resonance signals appeared at 5.34, 5.12, and 4.99 ppm, which were designated as residues A, B, and C, respectively [6,23,24]. Combined with the ^13^C (Figure 2b) and HSQC (Figure 2c) spectra, the corresponding resonances of anomeric carbons were ascribed to 107.57, 108.33, and 109.35 ppm. However, due to the limitation of the ^1^H NMR spectrum and the weak signals of the ^13^C NMR spectrum, 2D NMR spectra were applied. According to the COSY spectrum (Figure 2d), the signals assigned to protons of α-Ara*f*-(1→ residues were fully assigned, as confirmed by the TOCSY spectrum (Figure S4a). The corresponding carbon resonances were identified by HSQC experiments, as summarized in Table 3. A signal at 4.77/108.48 ppm was observed in the 2D NMR of the HSQC spectrum, which confirmed that residue D was of the α-configuration. When combined with the relative peak area percentage in methylation analysis, residue D was found to correspond to →3)-α-Ara*f*-(1→. Resonance signals at 75–87 ppm indicated C2~C4 of α-Ara*f* residues, and the resonance signal in the region of 61–62 ppm arose from C5 of α-Ara*f*-(1→ residue [25,26]. Along with the results of the COSY, TOCSY, and HSQC experiments, the other protons and carbons resonances of →3)-α-Ara*f*-(1→ residue are shown in Table 3. 

On the other hand, xylose (59.3%) was the main monosaccharide composition of HBAX-60 and comprised five linkage types: →2,3,4)-β-Xyl*p*-(1→ (residue E; 12.1%), →3,4)-β-Xyl*p*-(1→ (residue F; 5.9%), β-Xyl*p*-(1→ (residue G; 3.1%), →4)-β-Xyl*p*-(1→ (residue H; 36.2%), and →2,4)-β-Xyl*p*-(1→ (residue I; 1.2%). By referring to previous reports [6,23,24,26], main peaks in the anomeric regions of the 1D NMR spectra (^1^H and ^13^C NMR) could be easily identified, and their anomeric signals were assigned at 4.57/100.83, 4.32/102.18, 4.26/102.18, 4.24/102.18, and 4.40/103.96 ppm, respectively. They were interpretable to be →2,3,4)-β-Xyl*p*-(1→, →3,4)-β-Xyl*p*-(1→, β-Xyl*p*-(1→, →4)-β-Xyl*p*-(1→ and →2,4)-β-Xyl*p*-(1→. Resonance signals at 65–85 ppm corresponded to C2~C4 of β-Xyl*p* residues [25], while the chemical shifts in the region of 61–63 ppm corresponded to C5 of β-Xyl*p*-(1→ residues [26]. Moreover, most resonances of the cross peaks are summarized in Table 3 according to the 2D spectra (Figure 2c,d and Figure S4a). However, the signal of residue I was too low to be well-confirmed.

**Figure 3:**
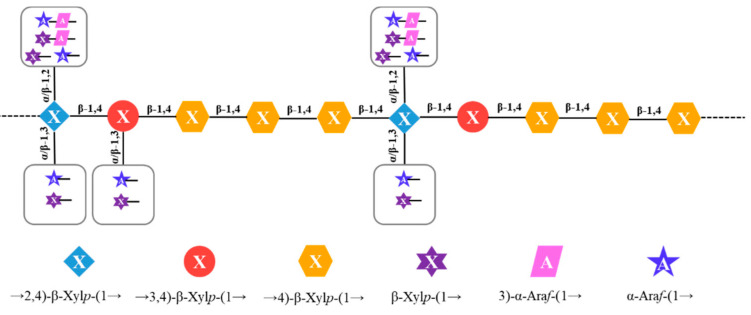
The proposed core units of arabinoxylan from HBAX-60.

In order to identify the sequence of glycosidic linkages existing in HBAX-60, long-range ^1^H and ^1^H correlation (NOESY) and ^1^H and ^13^C correlation (HMBC) spectra were generally preferred. Due to the low resolution and worthless spectral assignments of solvent peaks in the HMBC spectrum (not provided), the NOESY spectrum (Figure S4b) was able to identify the sequences of various bonds. From the cross peaks in the NOESY spectrum and relevant evidence, the long-range coupling between protons (residue H H1–residue H H4, residue F H1–residue H H4, residue F H1–residue E H4, and residue E H1–residue H H4) among xylose units implied that the *O*-1 of residue H was attached to the *O*-4 of residue H or residue E via β-(1→4) linkages [6,20,23,26]. Moreover, there were some signals observed for the anomeric protons from residues C and D attached to the H2 of residue E. Above all, the main chain of HBAX-60 was a low-branched arabinoxylan that possessed a →4)-β-Xyl*p*-(1→ (residue H) backbone linked by other branches. The possible linkages are summarized as follows [23,27,28,29]: (1) the terminal α-Ara*f*-(1→ or β-Xyl*p*-(1→ might be substituted at *O*-3 of →4)-β-Xyl*p*-(1→ residue, and (2) α-Ara*f*-(1→, β-Xyl*p*-(1→, β-Xyl*p*-(1→3)-α-Ara*f*-(1→ or α-Ara*f*-(1→3)-α-Ara*f*-(1→ might be substituted at the *O*-2 site, which still needs further identification. The →2)-α-Ara*f*-(1→ (1.4%), →5)-α-Ara*f*-(1→ (1.3%), Glc*p*-(1→ (0.8%), →3)-Glc*p*-(1→ (0.4%), and →4)-Glc*p*-(1→ (1.5%) residues could not be assigned because of their low relative peak area percentages. Finally, combined with the results from the above-mentioned GC–MS analysis and NMR experiments, the core unit structure of HBAX-60 was deduced, as shown in Figure 3.

### 2.5. Molecular Properties

**Figure 4:**
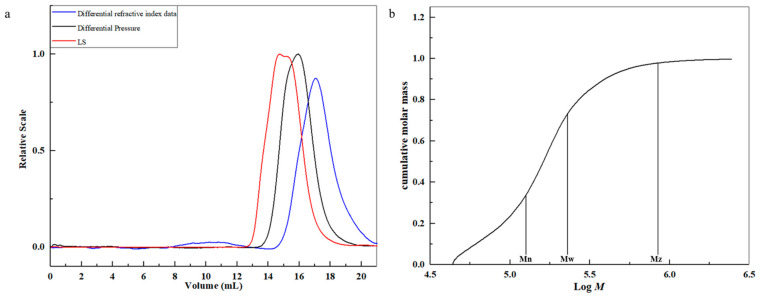

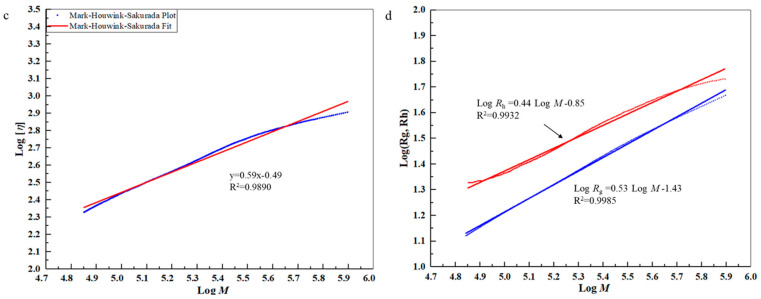
SEC elution profile of HBAX-60 (**a**); double logarithmic plots of *M*_w_ vs. cumulative weight fraction (**b**); [*η*] (**c**); and *R*_g_, *R*_h_, and the ratio of *R*_g_/*R*_h_ (**d**).

HBAX-60 had a high mean *M*_w_ of 222.3 kDa. The relationships of the mean molar number (*M*_n_) (129.3 kDa), *M*_w_, and *M*_z_ (800.6 kDa) with cumulative weight fraction are presented in Figure 4b. The value of *M*_w_/*M*_n_ of HBAX-60 was 1.7, indicating a relatively narrower distribution of arabinoxylan chains under the detected condition. 

The intrinsic viscosity ([*η*]), root mean square (*R*_g_), and average hydrodynamic radius (*R*_h_) are important parameters that can be applied to explore the solution properties of a polymer chain. According to the equation of Mark–Houwink–Sakurada (MHS) ([*η*] = *K*M*^α^*), the relationship of [*η*] (619.7 mL/g) and molecular mass could be associated with the “stiffness” of HBAX-60 [30]. The double logarithmic plots of *M*_w_ *versus* [*η*] showed a decreased slope (Figure 4c), revealing the presence of the enhancement of intermolecular interactions in the high *M*_w_ fraction. A major plot accounting for 84.0% of the total molecular mass was used to fit the curves to reflect more specific molecular properties. The value of *α* in the region was 0.59, which probably exhibited a random coil conformation in 0.1 mol/L NaNO_3_ solution [31]. The particle size distribution and aggregation state affected the viscosity of the solution and the structural network of the gel, as well as the functional properties of arabinoxylan. Through using linear regression (Figure 4d), the slope of Log *R*_g_ vs. Log *M*_w_ was found to be 0.44 and the slope of Log *R*_h_ vs. Log *M*_w_ was found to be 0.53, suggesting the presence of linear flexible polymer chains in aqueous solution [32]. Moreover, with the decrease of *M*_w_, the value of *R*_g_ and *R*_h_ decreased, which indicated that HBAX-60 had a better ductility in the 0.1 mol/L NaNO_3_ solution. 

## 3. Materials and Methods

### 3.1. Sample Preparation

Hull-less barley was cultivated in Qinghai Province, China. Enzymes (thermostable α-amylase, amyloglucosidase and papin) were provided by Aladdin Biochemical Technology Co., Ltd. (Shanghai, China). The β-glucan assay kit was from Megazyme International (Wicklow, Ireland). d-xylose was obtained from J&K Scientific Ltd. (Beijing, China). Other monosaccharide standards (d-arabinose, d-galactose, d-glucose, glucuronic acid, and galacturonic acid), deuterium oxide (D_2_O), dimethyl sulfoxide-d_6_ (DMSO-d_6_), methyl iodide, and sodium borodeuteride (98 atom% d) were bought from Sigma-Aldrich Chemical Co. (St. Louis, MO, USA). All other used reagents were of analytical grade unless otherwise specified.

### 3.2. Extraction and Purification of Arabinoxylan in Hull-Less Barley

**Figure 5:**
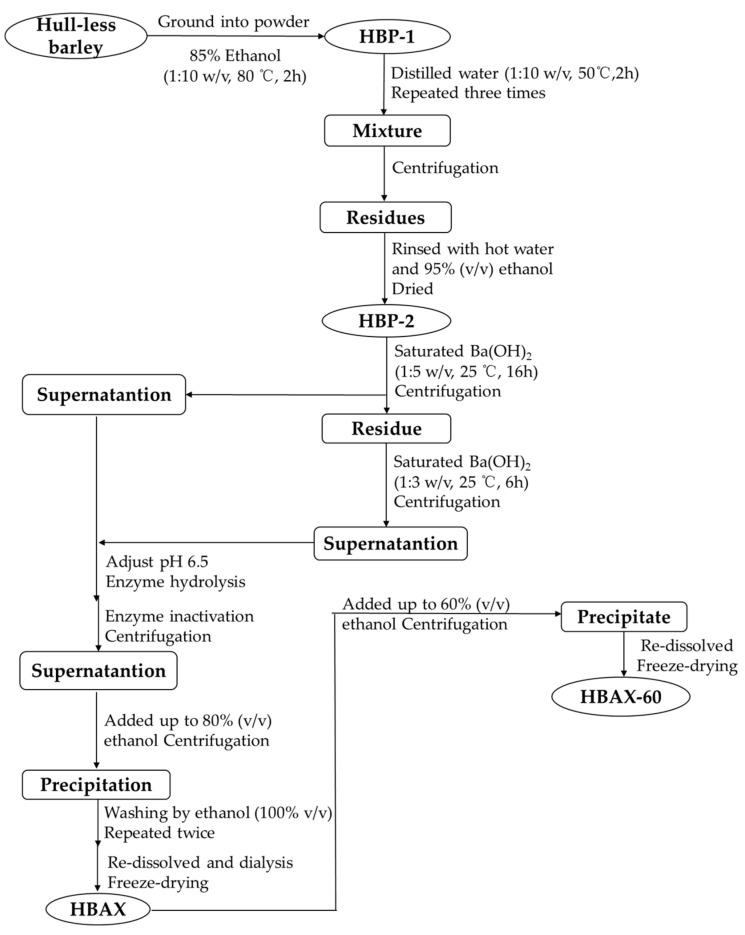
Extraction and purification processes of arabinoxylan from hull-less barley.

As shown in Figure 5, the whole hull-less barley grains were ground into powder (not sieved), which was then refluxed by 85% (*v*/*v*) ethanol for 2 h to remove oligosaccharides, volatile oil, and other small molecules [33]. The residue was separated and eventually dried to get a hull-less barley precipitate (HBP-1). Because arabinoxylan usually occurs together with β-glucan in cereal cell walls [34], HBP-1 was treated with hot water (1:10 *w*/*v*) three times to avoid the influence of β-glucan, followed by centrifugation to separate the residue (named as HBP-2) from the supernatant. Dried HBP-2 was extracted from a saturated barium hydroxide solution with constant stirring at 25 °C [20]. The extraction process was repeated twice. After centrifugation (5000 rpm for 15 min), the pH of the obtained supernatant was adjusted to be 6.5 with 2 mol/L HCl. Then, the supernatant was hydrolyzed by enzymes to successively remove starches and proteins, including α-amylase (95 °C for 3 h), amyloglucosidase (60 °C for 30 min), and papin (60 °C for 1 h). The enzymes were inactivated at 100 °C for 10 min and then centrifugated (5000 rpm for 15 min). The supernatant was concentrated, followed by an ethanol precipitation until a final concentration of 80% (*v*/*v*). The freeze-dried hull-less barley arabinoxylan (HBAX) was collected.

HBAX was dissolved to obtain a homogeneous aqueous solution (10%, *w*/*v*), and then 1.5 times volume of ethanol was added until the solution reach a final concentration of 60%. The fraction collected after centrifugation (10,000 rpm at 4 °C for 15 min) was washed with 100% ethanol and freeze-dried. The resultant fraction (HBAX-60) was chosen for further analysis.

### 3.3. Chemical Compositions

The content of neutral sugar was analyzed according to phenol-sulfuric acid method [35] with xylose as a standard, while the percentage of uronic acid was determined by the *m*-hydroxybiphenyl photometric assay with glucuronic acid as a standard [36]. Protein content was studied by a spectrophotometric method [37] with bovine serum albumin as the standard. The content of β-glucan was found with the Megazyme β-glucan assay kit (AOAC method 995.16, Wicklow, Ireland), and the determination of the total starch was conducted by the Megazyme total starch assay kit (AOAC method 996.11, Wicklow, Ireland). The content of arabinoxylan was identified with the orcinol–hydrochloric acid method [38]. In addition, monosaccharide composition was measured by following the method of a previous report [39] with some modifications. Polysaccharide (5 mg) was hydrolyzed in 12 mol/L H_2_SO_4_ in an ice-water bath for 30 min and then diluted into 2 mol/L in an oil bath at 100 °C for 2 h. The hydrolysate was diluted and passed through the hydrophilic membrane (0.22 μm), and then it was subjected to high-performance anion-exchange chromatography with pulsed amperometry (HPAEC-PAD).

The *M*_w_ distribution, radius of gyration, and [*η*] of HBAX-60 were quantified by high performance size exclusion chromatography (HPSEC) coupled with the three online detectors, as previously reported [30]. A Series 1500 pump (Scientific Systems Inc., Janesville, WI, USA) and two columns—SB-806 HQ column (8 × 300 mm) and SB-804 HQ column (8 × 300 mm, Showa Denko Inc., Tokyo, Japan)—in series were equipped in the HPSEC system, to which an SB-G guard column (6 × 50 mm) was connected. The eluent was a 0.1M NaNO_3_ solution (containing 0.02% NaN_3_) with a flow rate of 0.6 mL/min, while the columns were maintained at 35.0 ± 0.5 °C. HBAX-60 was injected after passing through a 0.22 μm hydrophilic membrane. All parameters were processed and analyzed using the ASTRA 6.1.7 software (Wyatt Technology Corporation, Santa Barbara, CA, USA). A refractive index increment (d*n*/d*c*) of 0.146 mL/g was used in the calculation.

### 3.4. SEM and FT-IR Spectroscopy Analysis

The HBAX-60 re-dissolved in distilled water (1.0 mg/mL) was freeze-dried and placed onto an aluminum stub and gold-coated with an ion sputter coater with an SU8100 SEM (Hitachi Ltd., Tokyo, Japan) for observation.

The polysaccharide sample, mixed with KBr powder, was ground and pressed as tablets prior to FT-IR spectroscopy, which was conducted with a Thermo Nicolet 5700 infrared spectrograph (Thermo-Electron, Madison, WI, USA) in a range from 4000 to 400 cm^−1^. In addition, each FT-IR spectrum was scanned 32 times at a resolution ratio of 4 cm^−1^.

### 3.5. Methylation and GC–MS Analysis

The assignments of linkage types in HBAX-60 were analyzed with GC–MS according to the method of Shi et al. [31]. A dried polysaccharide sample (2–3 mg) was dissolved in anhydrous DMSO and methylated with 1 mL of iodomethane. The methylated sample was converted into partially methylated alditol acetates (PMAAs) by hydrolysis and acetylation. Then, the acetylated sample was injected into an Agilent Technology 7890/7000 QQQ system (Agilent Technologies Corp., Santa Clara, CA, USA) equipped with a Supelco-2330 column (30 m × 0.25 mml 0.2 μm film thickness).

### 3.6. NMR Analysis

Freeze-dried HBAX-60 was exchanged with D_2_O (5:1 *w*/*v*) three times and then dissolved in DMSO*-d*_6_ before testing. NMR experiments were conducted on a Bruker Avance III HD 400 MHz NMR spectrometer (Brucker, Switzerland) coupled with a liquid nitrogen cryogenic probe (CryoProbe^TM^ Prodigy, 5 mm BB_0_, Fällanden, Switzerland). The ^1^H, ^13^C, ^1^H-^1^H homonuclear correlated spectroscopy (^1^H-^1^H COSY), total correlated spectroscopy (TOCSY), nuclear Overhauser effect spectroscopy (NOESY), ^1^H-^13^C heteronuclear single-quantum coherence (^1^H-^13^C HSQC), and heteronuclear multiple-bond correlation (HMBC) spectra were recorded at 303 K with the standard Bruker pulse sequence.

## 4. Conclusions

In this work, highly pure arabinoxylan (HBAX-60) was isolated from the whole grain of hull-less barley from an alkaline solution and purified through enzymatic hydrolysis and ethanol precipitation. HBAX-60 was considered to be a relatively low-branched polysaccharide comprising un-substituted (1,4-linked β-Xyl*p*, 36.2%), mono-substituted (1,3,4-linked β-Xyl*p*, 5.9%), and di-substituted (1,2,3,4-linked β-Xyl*p*, 12.1%) xylose as the backbone, as well as some terminal α-Ara*f*, β-Xyl*p* and 1,3-linked Ara*f* as the side chains. HBAX-60 presented a spherical shape with a filamentary or linear appearance in solid morphology. HBAX-60 exhibited random coil conformation in a 0.1 M NaNO_3_ solution, which could provide a basis for studying conformational behaviors in different aqueous systems in other ways, such as dynamic and static light scattering. Branches of AX have positive effects on film formation and properties [40], which could be discussed in further research focusing on the amount and positions of branching in HBAX-60. This study provides a foundation for potential applications in food and other value-added health products of hull-less barley. How highly purified HBAX-60′s fine structure affects its functional characteristics and biological activities needs further study.

## Figures and Tables

**Table 1:** Chemical properties and monosaccharide compositions of arabinoxylan from hull-less barley.

Sample	Yield (%)	Neutral Sugar (%)	Uronic Acid (%)	Protein (%)	Starch (%)	β-Glucan (%)	AX (%)	Monosaccharide Composition (%, *w*/*w*) ^c^				A/X Ratio
								Ara	Gal	Glc	Xyl	
HBAX	1.5 ^a^	83.6 ± 1.8	3.5 ± 0.6	10.2 ± 0.1	0.4 ± 0.2	1.6 ± 0.1	67.9 ± 2.2	26.3 ± 2.2	0.9 ± 0.1	5.1 ± 0.1	38.3 ± 0.6	0.7
HBAX-60	72.2 ^b^	98.3 ± 1.4	n.d.	7.0 ± 0.1	0.3 ± 0.1	n.d.	93.7 ± 3.0	40.7 ± 2.0	n.d.	0.1 ± 0.1	59.3 ± 6.6	0.7

Ara: arabinose; Gal: galactose; Glc: glucose; Xyl: xylose; n.d.: not detected or lower than limit of quantification. ^a^ Yield of subfractions (%, *w*/*w*) based on the amount of material recovered after fractionation (HBP-2). ^b^ Yield of subfractions (%, *w*/*w*) based on the amount of HBAX. ^c^ The content was calculated based on the amount of the sample measured—the amount of individual monosaccharide detected by HPAEC/the weight of HBAX-60 × 100%.

**Table 2:** Chemical properties and monosaccharide compositions of arabinoxylan from hull-less barley.

Mass Fragments (*m*/*z*)	PMAAs ^a^	Linkage Pattern	Relative Peak Area Percentage (%) ^b^
43,101,102,117,118,161,162	1,5-di-*O*-acetyl-2,3,4-tri-*O*-methyl xylitol	Xyl*p*-(1→	3.1
43,87,102,118,129,162,189,233	1,4,5-tri-*O*-acetyl-2,3-di-*O*-methyl xylitol	→4)-Xyl*p*-(1→	36.2
43,87,88,129,130,189,190,234	1,2,4,5-tera-*O*-acetyl-3-*O*-methyl xylitol	→2,4)-Xyl*p*-(1→	1.2
43,59,85,118,160,201,261	1,3,4,5-tri-*O*-acetyl-2-*O*-methyl xylitol	→3,4)-Xyl*p*-(1→	5.9
43,73,74,115,116,145,146,217,218,289,290	1,2,3,4,5-penta-*O*-acetyl-xylitol	→2,3,4)-Xyl*p-*(1→	12.1
		Total	58.5
43,87,101,102,118,129,145,161,162,205	1,4-di-*O*-acetyl-2,3,5-tri-*O*-methyl arabinitol	Ara*f*-(1→	34.4
43,87,88,101,129,130,161,190	1,2,4-tri-*O*-acetyl-3,5-di-*O*-methyl arabinitol	→2)-Ara*f*-(1→	1.4
43,59,87,113,118,160,202,233	1,3,4-tri-*O*-acetyl-2,5-di-*O*-methyl arabinitol	→3)-Ara*f*-(1→	1.7
43,59,87,102,117,118,129,189,234	1,4,5-tri-*O*-acetyl-2,3-di-*O*-methyl arabinitol	→5)-Ara*f*-(1→	1.3
		Total	38.8
43,87,102,118,129,145,161,162,205	1,5-di-*O*-acetyl-2,3,4,6-tetra-*O*-methyl glucitol	Glc*p*-(1→	0.8
43,87,101,118,129,161,234	1,3,5-tri-*O*-acetyl-2,4,6-tri-*O*-methyl glucitol	→3)-Glc*p*-(1→	0.4
43,87,118,129,162,233	1,4,5-tri-*O*-acetyl-2,3,6-tri-*O*-methyl glucitol	→4)-Glc*p*-(1→	1.5
		Total	2.7

^a^ Determined from mass spectra of the PMAAs in Figure S3. The PMAAs were derived from individual sugars, e.g., 1,5-di-*O*-acetyl-2,3,4-tri-*O*-methyl xylitol means that xylose was methylated at the *O*-2,3,4 sites and acetylated at the *O*-1,5 sites, which indicates that the corresponding linkage pattern was Xyl*p*-(1→. ^b^ Ratio of each sugar residue was based on the percentage of its peak area.

**Table 3:** Assignments of ^1^H and ^13^C NMR chemical shifts of HBAX-60.

Code	Residue Linkages	H1/C1	H2/C2	H3/C3	H4/C4	H5/C5
A	α-Ara*f*-(1→3 ^a^	5.34/107.57	3.88/82.00	3.65/77.85	3.96/86.73	3.44/61.49
B	α-Ara*f*-(1→3 ^b^	5.12/108.33	3.82/80.45	3.68/78.03	3.84/85.19	3.45/61.60
C	α-Ara*f*-(1→2 ^c^	4.99/109.35	3.27/75.67	3.49/76.15	3.85/84.68	-
D	→3)-α-Ara*f*-(1→	4.77/108.48	3.43/76.07	-	-	-
E	→2,3,4)-β-Xyl*p*-(1→	4.57/100.83	3.70/78.03	3.41/75.08	3.83/75.83	3.44/61.26
F	→3,4)-β-Xyl*p*-(1→	4.32/102.18	3.05/73.38	3.26/76.97	-	-/61.76
G	β-Xyl*p*-(1→	4.26/102.18	3.04/73.54	-	-	-/61.92
H	→4)-β-Xyl*p*-(1→	4.24/102.18	3.03/73.22	3.25/74.44	3.62/73.70	3.32/63.30

“-“ means not detected. ^a^ The α-Ara*f*-(1→ residue was attached to *O*-3-mono-substituted β-Xyl*p* residues. ^b^ The α-Ara*f*-(1→ residue was attached to *O*-3-di-substituted β-Xyl*p* residues. ^c^ The α-Ara*f*-(1→ residue was attached to *O*-2-di-substituted β-Xyl*p* residues.

## Data Availability

Not applicable.

## Supplementary Materials

The following are available online. Figure S1. SEM images of HBAX in hull-less barley: (a) 3000 × and (b) 5000 ×; Figure S2. FT-IR spectrum of HBAX in hull-less barley; Figure S3. The total ion chromatography of PMAA derivatives (a) and mass spectra (b–m) of HBAX-60; b: 1,4-di-*O*-acetyl-2,3,5-tri-*O*-methyl arabinitol; c: 1,5-di-*O*-acetyl-2,3,4-tri-*O*-methyl xylitol; d: 1,2,4-tri-*O*-acetyl-3,5-di-*O*-methyl arabinitol; e: 1,5-di-*O*-acetyl-2,3,4,6-tetra-*O*-methyl glucitol; f: 1,3,4-tri-*O*-acetyl-2,5-di-*O*-methyl arabinitol; g: 1,4,5-tri-*O*-acetyl-2,3-di-*O*-methyl arabinitol; h: 1,4,5-tri-*O*-acetyl-2,3-di-*O*-methyl xylitol; i: 1,3,5-tri-*O*-acetyl-2,4,6-tri-*O*-methyl glucitol; j: 1,4,5-tri-*O*-acetyl-2,3,6-tri-*O*-methyl glucitol; k: 1,2,4,5-tera-*O*-acetyl-3-*O*-methyl xylitol; l: 1,3,4,5-tri-*O*-acetyl-2-*O*-methyl xylitol; m: 1,2,3,4,5-penta-*O*-acetyl-xylitol. The PMAAs were derived from individual sugars, e.g., 1,5-di-*O*-acetyl-2,3,4-tri-*O*-methyl xylitol means that xylose was methylated at the *O*-2,3,4 sites and acetylated at the *O*-1,5 sites, which indicates that the corresponding linkage pattern was Xyl*p*-(1→. Figure S4. TOCSY (**a**) and NOESY (**b**) NMR spectra of HBAX-60.

## Sample Availability

Samples of the compounds are available from the authors.
