# Supplementary material for: Isolation, Physicochemical Properties, and Structural Characteristics of Arabinoxylan from Hull-Less Barley
Source: Molecules. 2021 May 19;26(10):3026. doi: 10.3390/molecules26103026 (PMC8161004; doi:10.3390/molecules26103026)
Supplement: Supplementary file 1 [file molecules-26-03026-s001.zip › molecules-1206979-supplementary.pdf]

Supplemental Data

Figure S1. SEM images of HBAX in hull-less barley: a) 3000 × and b) 5000 ×.

Figure S2. FT-IR spectrum of HBAX in hull-less barley.

Figure S3. The total ion chromatography of PMAAs derivatives (a) and mass spectra (b–m) of HBAX-60. b: 1,4-di-*O*-acetyl-2,3,5-tri-*O*-methyl arabinitol; c: 1,5-di-*O*-acetyl-2,3,4-tri-*O*-methyl xylitol; d: 1,2,4-tri-*O*-acetyl-3,5-di-*O*-methyl arabinitol; e: 1,5-di-*O*-acetyl-2,3,4,6-tetra-*O*-methyl glucitol; f: 1,3,4-tri-*O*-acetyl-2,5-di-*O*-methyl arabinitol; g: 1,4,5-tri-*O*-acetyl-2,3-di-*O*-methyl arabinitol; h: 1,4,5-tri-*O*-acetyl-2,3-di-*O*-methyl xylitol; i: 1,3,5-tri-*O*-acetyl-2,4,6-tri-*O*-methyl glucitol; j: 1,4,5-tri-*O*-acetyl-2,3,6-tri-*O*-methyl glucitol; k: 1,2,4,5-tera-*O*-acetyl-3-*O*-methyl xylitol; l: 1,3,4,5-tri-*O*-acetyl-2-*O*-methyl xylitol; and m: 1,2,3,4,5-penta-*O*-acetyl-xylitol. The PMAAs were derived from individual sugars, e.g., 1,5-di-*O*-acetyl-2,3,4-tri-*O*-methyl xylitol means that xylose was methylated at the *O*-2,3,4 sites and acetyled at the *O*-1,5 sites, which indicates that the corresponding linkage pattern was Xyl*p*-(1→.

Figure S4. TOCSY (a) and NOESY (b) spectra of HBAX-60.

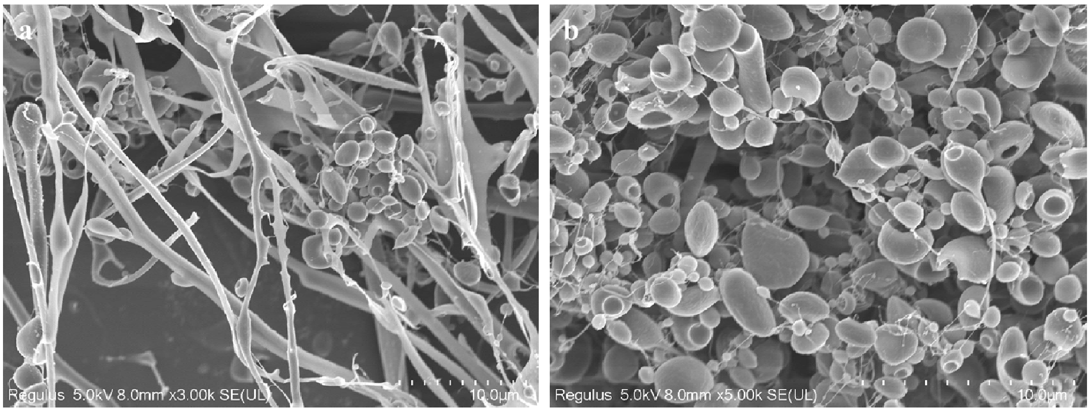

Figure S1. SEM images of HBAX in hull-less barley: (**a**) 3000 × and (**b**) 5000 ×.

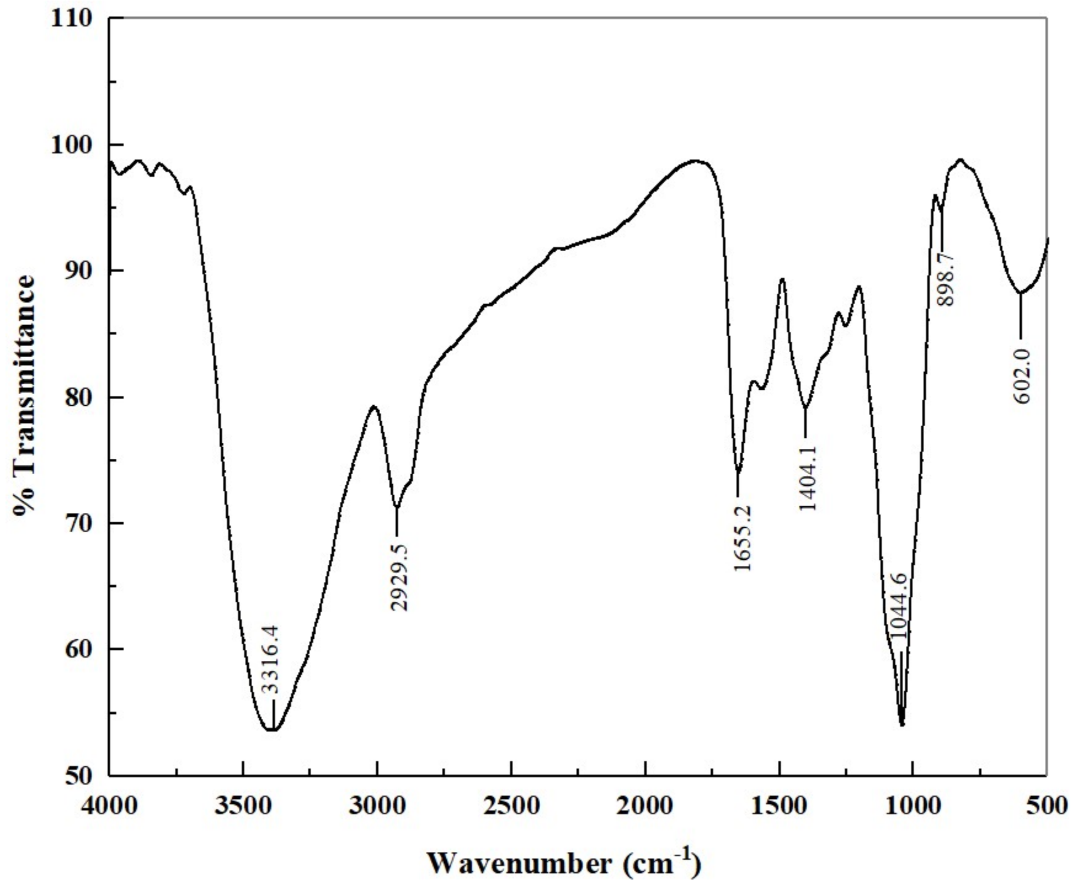

Figure S2. FT-IR spectrum of HBAX in hull-less barley.

a

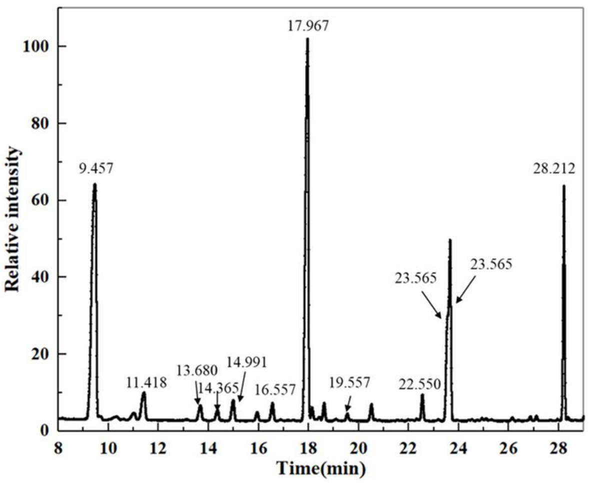
Relative intensity
Time(min)
9.457
11.418
13.680
14.365
14.991
16.557
17.967
19.557
22.550
23.565
23.565
28.212

b

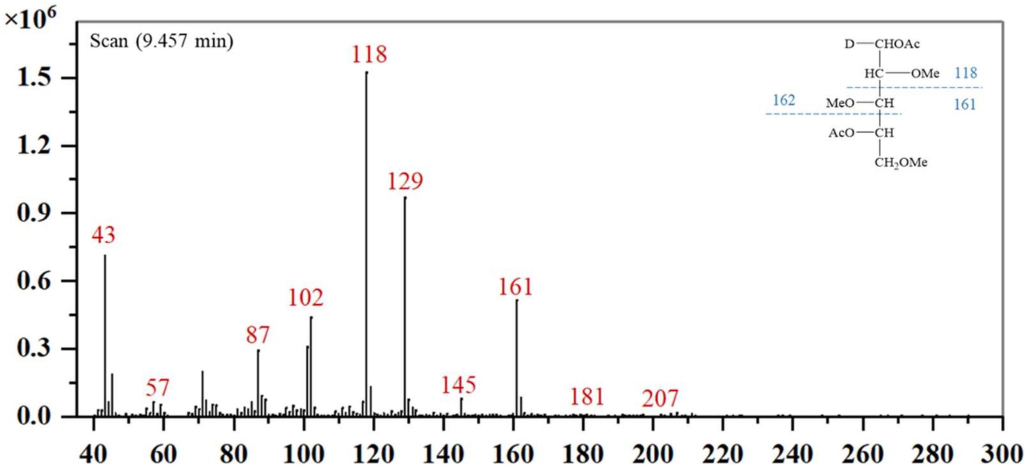
×10^6
Scan (9.457 min)
43
57
87
102
118
129
145
161
181
207
D—CHOAc
HC—OMe
MeO—CH
AcO—CH
CH₂OMe
118
162
161

c

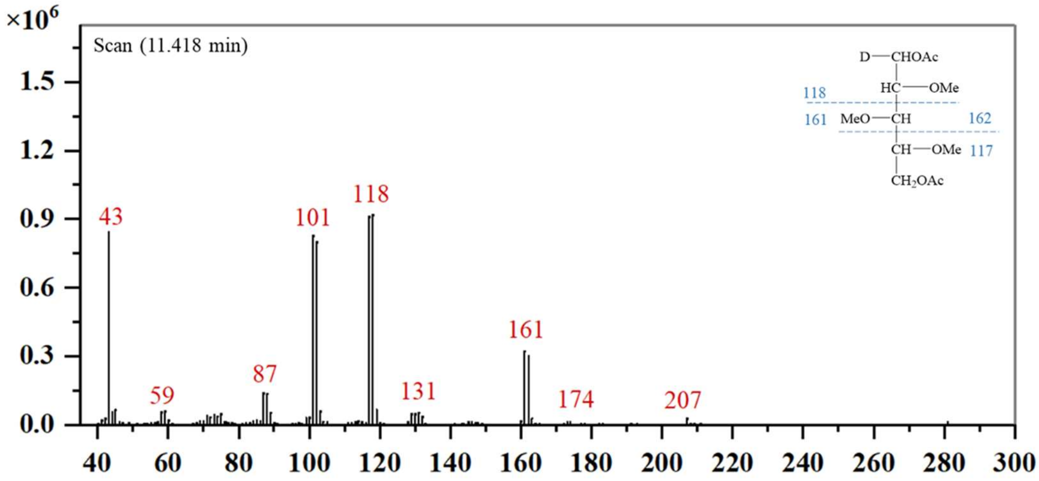
×10^6
Scan (11.418 min)
43
59
87
101
118
131
161
174
207
D—CHOAc
HC—OMe
MeO—CH
CH—OMe
CH₂OAc
118
161
162
117

d

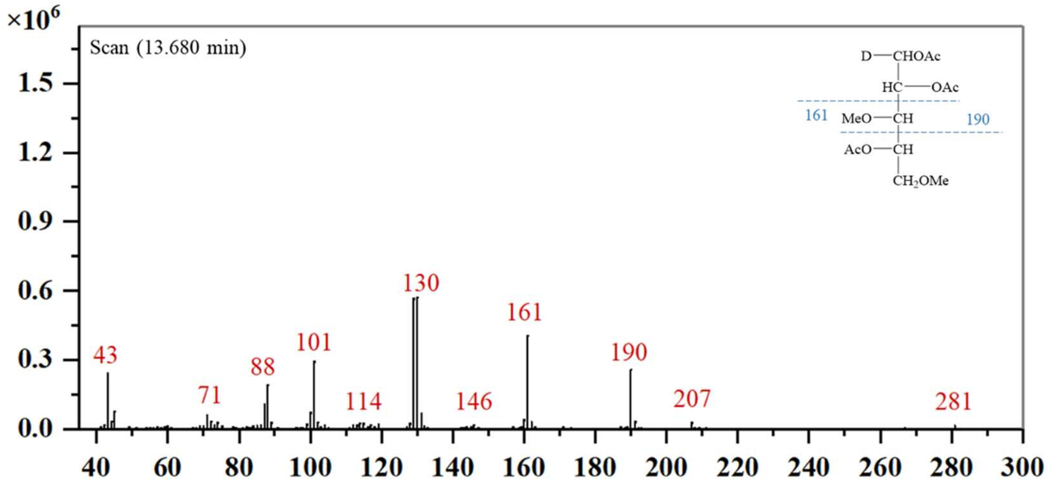
×10^6
Scan (13.680 min)
D—CHOAc
HC—OAc
161
MeO—CH
190
AcO—CH
CH2OMe
43
71
88
101
114
130
146
161
190
207
281
0.0 0.3 0.6 0.9 1.2 1.5
40 60 80 100 120 140 160 180 200 220 240 260 280 300

e

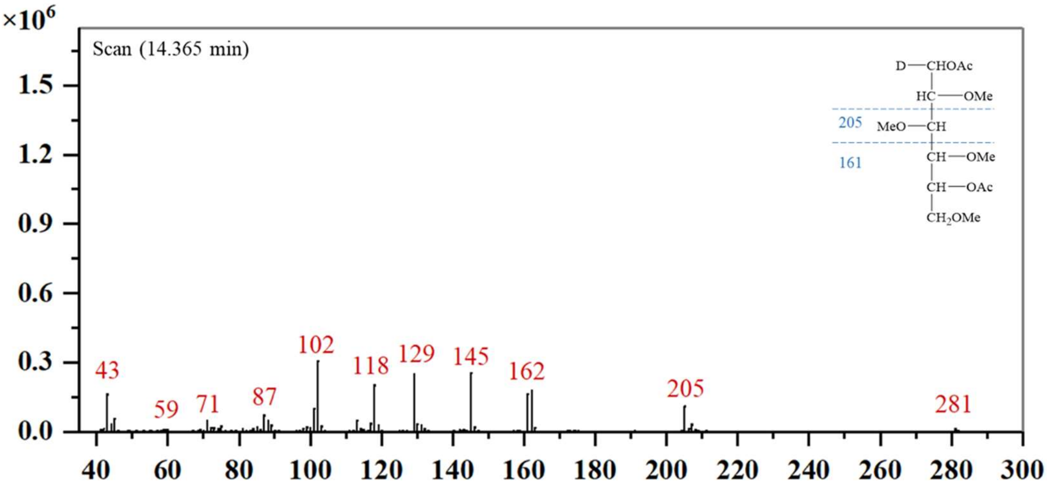
×10^6
Scan (14.365 min)
D—CHOAc
HC—OMe
205
MeO—CH
161
CH—OMe
CH—OAc
CH2OMe
43
59
71
87
102
118
129
145
162
205
281
0.0 0.3 0.6 0.9 1.2 1.5
40 60 80 100 120 140 160 180 200 220 240 260 280 300

f

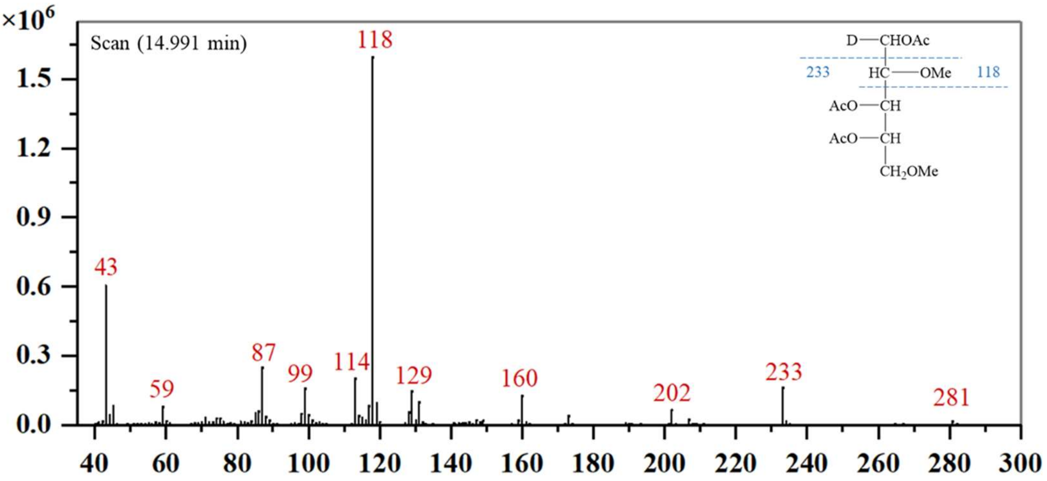
×10^6
Scan (14.991 min)
D—CHOAc
233
HC—OMe
118
AcO—CH
AcO—CH
CH2OMe
43
59
87
99
114
118
129
160
202
233
281
0.0 0.3 0.6 0.9 1.2 1.5
40 60 80 100 120 140 160 180 200 220 240 260 280 300

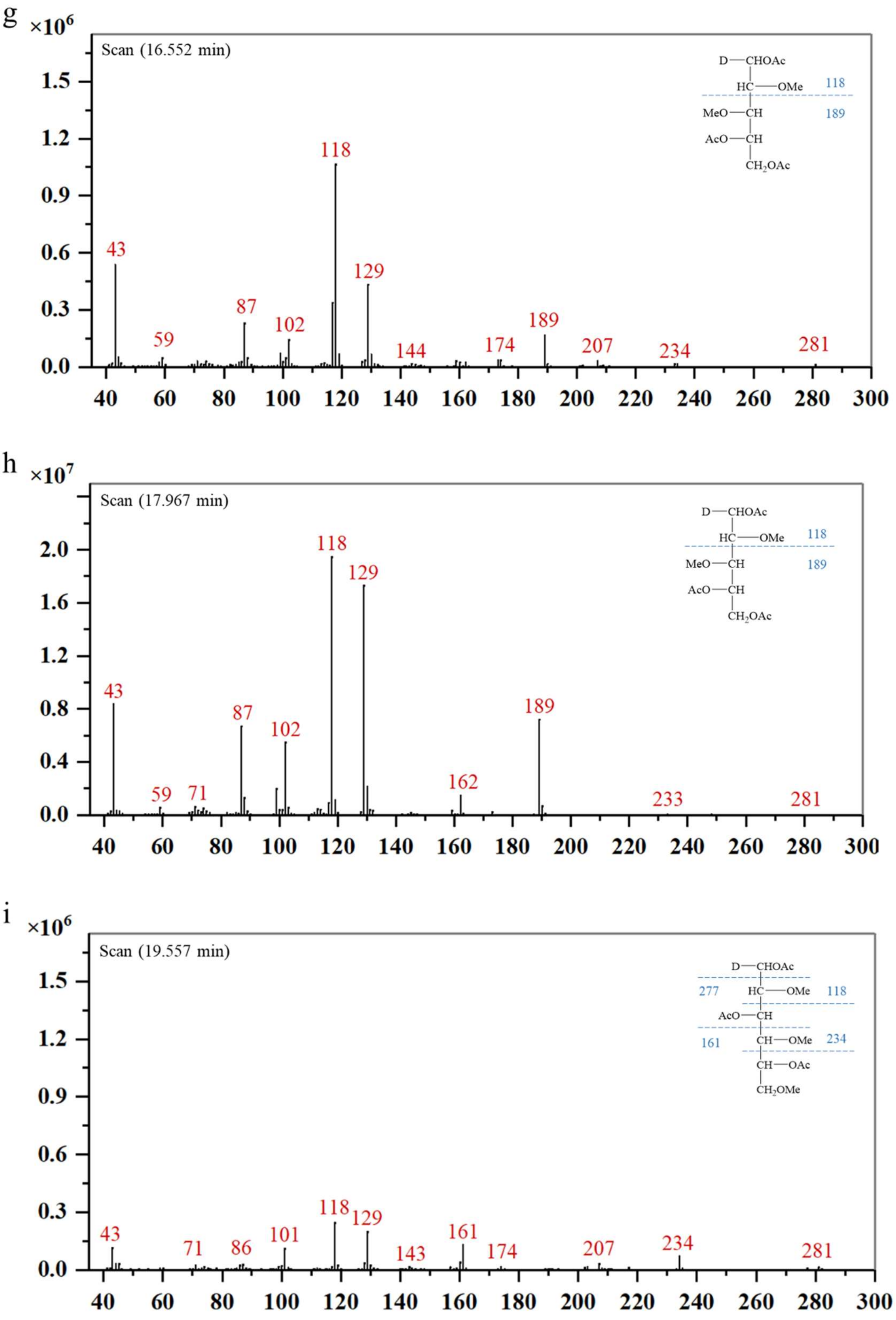
g
×10^6
Scan (16.552 min)
D—CHOAc
HC—OMe
MeO—CH
AcO—CH
CH2OAc
118
189
43
59
87
102
118
129
144
174
189
207
234
281
h
×10^7
Scan (17.967 min)
D—CHOAc
HC—OMe
MeO—CH
AcO—CH
CH2OAc
118
189
43
59
71
87
102
118
129
162
189
233
281
i
×10^6
Scan (19.557 min)
D—CHOAc
HC—OMe
AcO—CH
CH—OMe
CH—OAc
CH2OMe
277
118
161
234
43
71
86
101
118
129
143
161
174
207
234
281

j

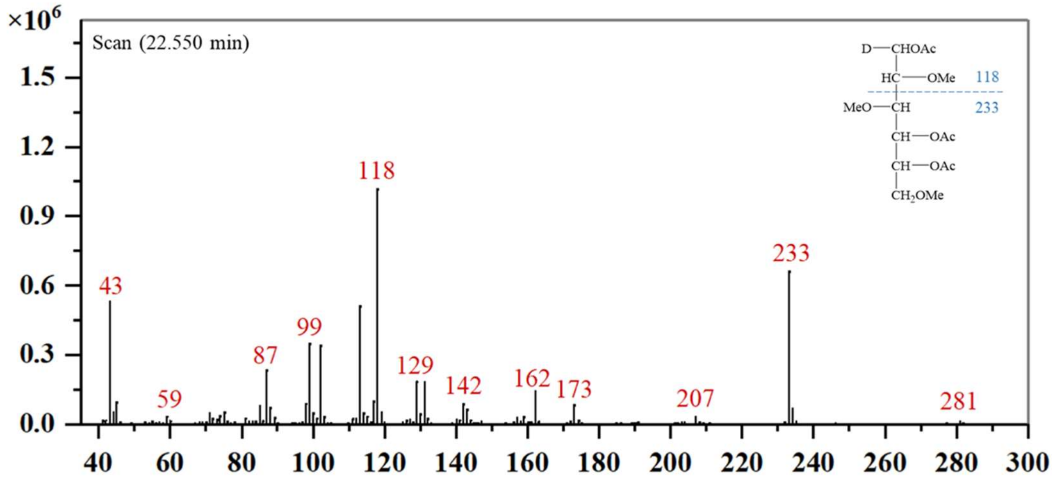
×10^6
Scan (22.550 min)
D—CHOAc
HC—OMe 118
MeO—CH 233
CH—OAc
CH—OAc
CH2OMe
118
233
43
99
87
129
59
142
162
173
207
281

k

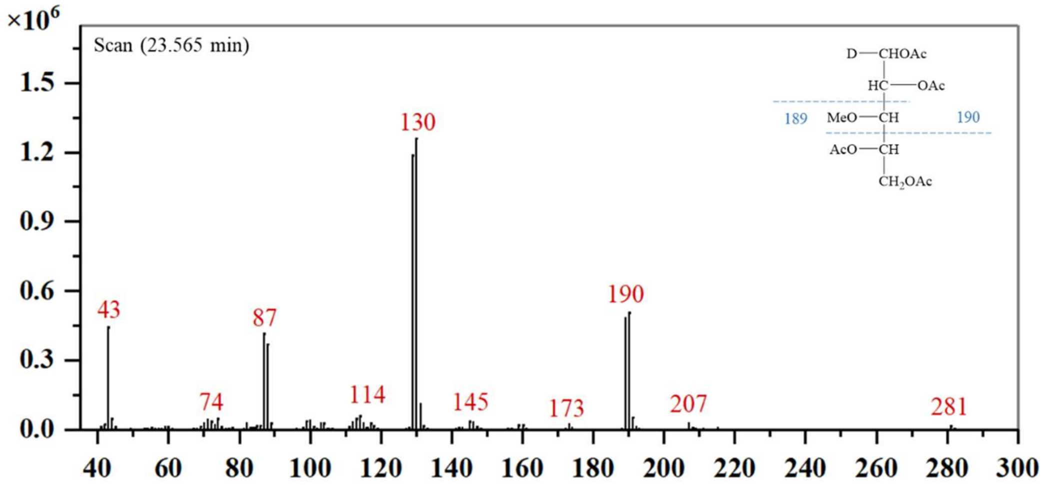
×10^6
Scan (23.565 min)
D—CHOAc
HC—OAc
189 MeO—CH 190
AcO—CH
CH2OAc
130
190
43
87
74
114
145
173
207
281

l

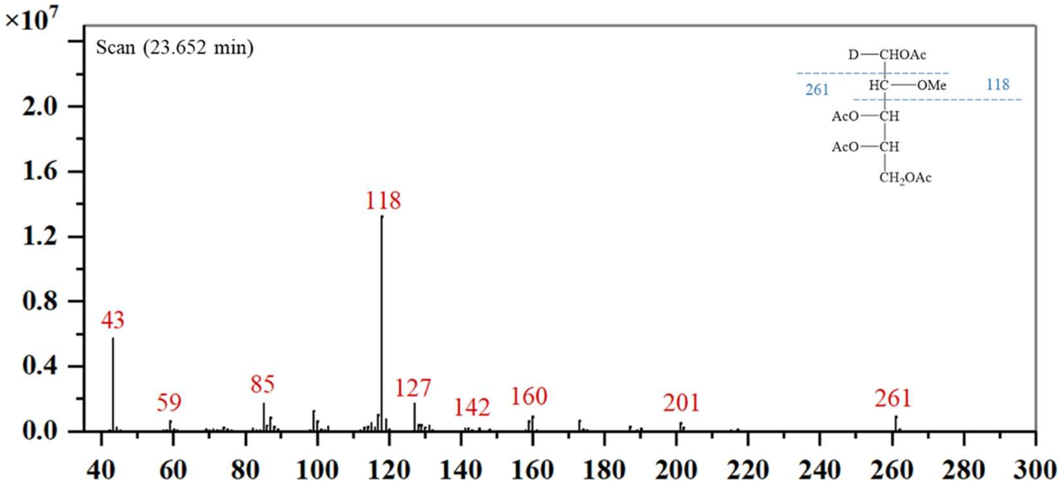
×10^7
Scan (23.652 min)
D—CHOAc
261 HC—OMe 118
AcO—CH
AcO—CH
CH2OAc
118
43
59
85
127
142
160
201
261

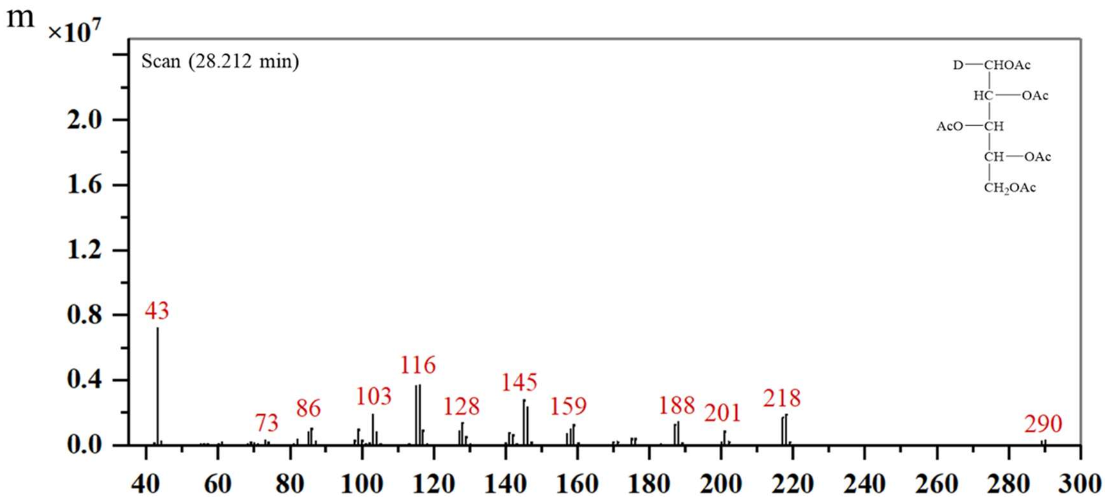

Figure S3. The results of methylation analysis. (**a**) Total ion chromatography of the PMAAs derivatives; (**b**–**m**) mass fragments of each linkage pattern: (**b**) 1,4-di-*O*-acetyl-2,3,5-tri-*O*-methyl arabinitol; (**c**) 1,5-di-*O*-acetyl-2,3,4-tri-*O*-methyl xylitol; (**d**) 1,2,4-tri-*O*-acetyl-3,5-di-*O*-methyl arabinitol; (**e**) 1,5-di-*O*-acetyl-2,3,4,6-tetra-*O*-methyl glucitol; (**f**) 1,3,4-tri-*O*-acetyl-2,5-di-*O*-methyl arabinitol; (**g**) 1,4,5-tri-*O*-acetyl-2,3-di-*O*-methyl arabinitol; (**h**) 1,4,5-tri-*O*-acetyl-2,3-di-*O*-methyl xylitol; (**i**) 1,3,5-tri-*O*-acetyl-2,4,6-tri-*O*-methyl glucitol; (**j**) 1,4,5-tri-*O*-acetyl-2,3,6-tri-*O*-methyl glucitol; (**k**) 1,2,4,5-tera-*O*-acetyl-3-*O*-methyl xylitol; (**l**) 1,3,4,5-tri-*O*-acetyl-2-*O*-methyl xylitol; (**m**): 1,2,3,4,5-penta-*O*-acetyl-xylitol. The PMAAs were derived from individual sugars, e.g., 1,5-di-*O*-acetyl-2,3,4-tri-*O*-methyl xylitol means that xylose was methylated at the *O*-2,3,4 sites and acetyled at the *O*-1,5 sites, which indicates that the corresponding linkage pattern was Xyl*p*-(1→.

a

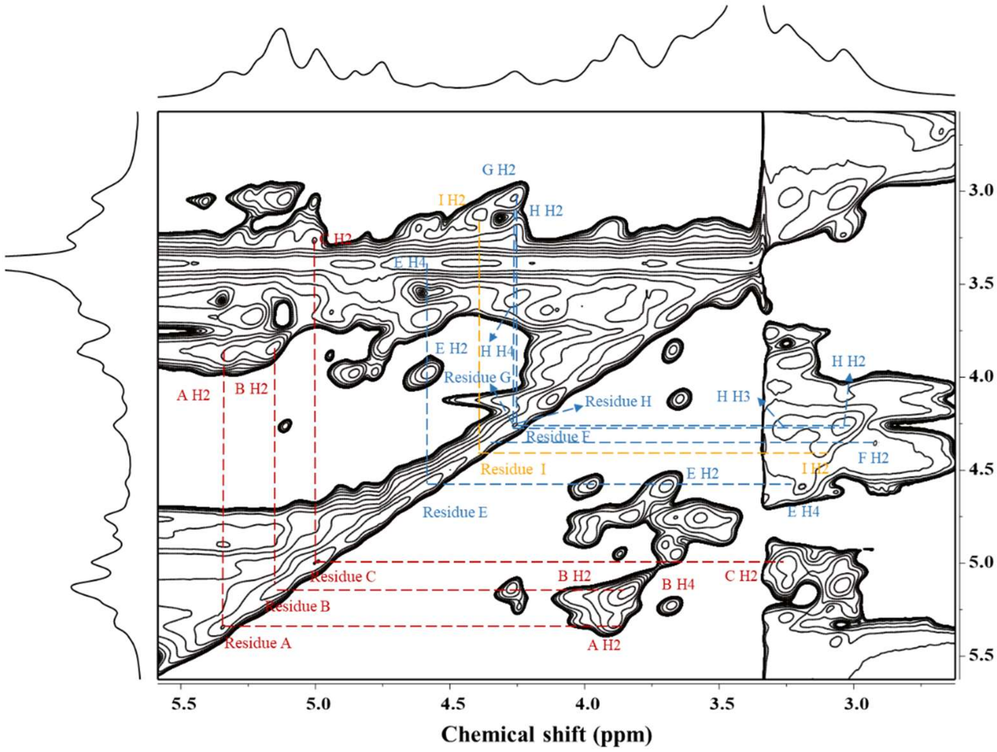

b

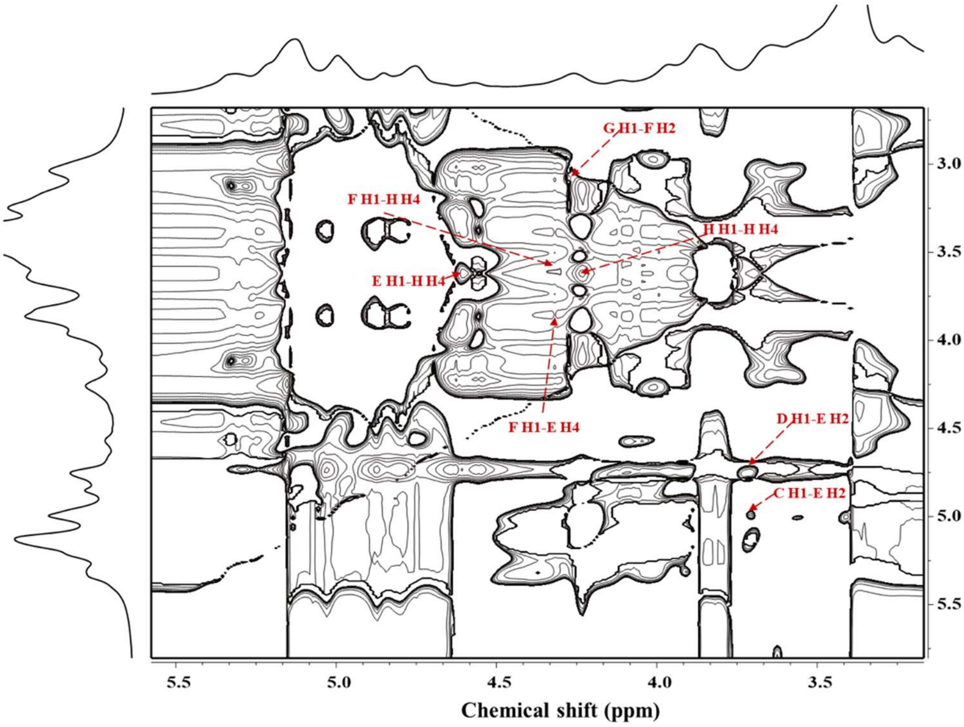

Figure S4. NMR spectra of HBAX-60: (**a**) TOCSY NMR spectrum; (**b**) NOESY NMR spectrum.
